# A Systematic Review of the Correlation Between Micronutrient Levels and Perinatal Depression

**DOI:** 10.3390/nu17213479

**Published:** 2025-11-05

**Authors:** Nabilah Islam, Annalese Semmler, Jean Starling, Joanne Voisey

**Affiliations:** 1 Centre for Genomics and Personalised Health, School of Biomedical Sciences, Faculty of Health, Queensland University of Technology, Kelvin Grove, QLD 4059, Australia; nabilah.islam@hdr.qut.edu.au; 2 School of Clinical Sciences, Faculty of Health, Queensland University of Technology, Kelvin Grove, QLD 4059, Australia; annalese.semmler@qut.edu.au; 3 Campbelltown Hospital, Ambarvale, NSW 2560, Australia; jean.starling@health.nsw.gov.au

**Keywords:** perinatal depression, micronutrients, vitamin D, maternal mental health, biomarkers, postpartum depression

## Abstract

Background: Depression is a significant complication of the peripartum period that can result in profound long-term detrimental implications for the affected woman, her child, and her family. It is possible that micronutrient imbalances could contribute to the development of perinatal depression through their roles in neurotransmitter synthesis and neuroendocrine and neuroimmune pathways. Micronutrient imbalances are more likely during the perinatal period due to the additional physiological demands on the body during this time. The objective of this systematic review was to review and summarise the existing evidence regarding the association between micronutrient levels and perinatal depression. Methods: MEDLINE, EMBASE, PsycINFO, CINAHL, Scopus, and Web of Science were searched for studies examining blood levels of micronutrients and assessment of depression within the peripartum period using validated rating tools. Results: A total of 58 studies met the eligibility criteria and were included in this review. Of these, 31 studies reported a significant inverse association between perinatal depression and at least one of the following: vitamin D, iron status, vitamin B12, folate, or zinc. Vitamin D was the most frequently investigated nutrient, examined in 28 of the 58 articles. The remaining 27 did not demonstrate a significant association. Conclusion: This review found that vitamin D deficiency has the greatest evidence of an association with perinatal depression. The evidence for other micronutrients is mixed, inconclusive, or limited. Further research is required to determine the significance of these micronutrients in the development of perinatal depression.

## 1. Introduction

Perinatal depression is a significant complication of pregnancy and the postpartum period. One study estimated the world-wide prevalence of perinatal depression to be 11.98% (95% CI 11.4–12.5) [1]. Perinatal (or peripartum) depression refers to depression arising during pregnancy (antenatal period) or after childbirth, up to 12 months postpartum [2]. Peripartum depression can have profound long-term detrimental implications for the affected woman, her child, and her family. The UK-based confidential enquiry into maternal deaths study found suicide to be the leading cause of maternal death during the postpartum period [3]. Depression during pregnancy is associated with reduced prenatal care, increased maternal substance use, and higher rates of premature delivery and small-for-gestational-age (SGA) infants [4]. Postpartum depression (PPD) is associated with problems with maternal behaviour and interpersonal relations, including difficulty with infant care and other responsibilities, and difficulty bonding with the child and marital relationship issues [5]. Importantly, perinatal depression may have lasting effects on the child’s development [6].

The current literature indicates that several micronutrients may be implicated in the development of perinatal depression. This is plausible considering that a number of micronutrients play important roles in brain neurochemistry. Decreased brain iron stores, for example, impair the activity of iron-dependent enzymes that are necessary for the synthesis of serotonin, dopamine, and noradrenaline [7]. Vitamin B12 and folate are linked to the effective functioning of the folate cycle, which is necessary for the regeneration of tetrahydrobiopterin, a co-factor that has a crucial role in the synthesis of neurotransmitters [8]. Zinc has a key role in neurotransmitter actions, including serotonin, the functioning of GABA and NMDA receptors, and the activation of neurotrophic factors [9]. Particularly, during the perinatal period, women are more vulnerable to developing micronutrient imbalances due to the increased physiological needs of the body [10]. Thus, our hypothesis is that micronutrient imbalances contribute to the development of perinatal depression. Current Australian guidelines informing clinical practice for perinatal mental illness do not provide any recommendations regarding pathology testing for perinatal depression [11,12,13,14]. Understanding the impact of micronutrient deficiencies during the perinatal period may be able to help inform current clinical practice with regard to the testing and management of these micronutrient imbalances to help in the prevention or treatment of perinatal depression.

## 2. Methods

### 2.1. Search Strategy

This systematic review followed the Preferred Reporting Items for Systematic Reviews and Meta-analyses (PRISMA) guidelines. The protocol is registered in PROSPERO (CRD42024620320) and is accessible at https://www.crd.york.ac.uk/PROSPERO/view/CRD42024620320 (accessed on 30 October 2025). A comprehensive search of the electronic databases (Ovid databases, MEDLINE, EMBASE, PsycINFO, CINAHL, Scopus, and Web of Science) was undertaken from the time of inception on 14 June 2024.

Two main searches were performed as described below and then combined with the word “AND” to produce the search for each search engine. Each of the following terms was searched as keywords on their own and as subject headings when possible:

1. “Postpartum depression” OR “Postnatal depression” OR “Antepartum depression” OR “Antenatal depression: OR “Peripartum depression” OR “Perinatal depression”
2. “Micronutrient*” OR “Biological marker*” OR “Biomarker*” OR “Trace element*” OR “Trace metal*” OR “Zinc” OR “Copper” OR “Selenium” OR “Magnesium” OR “Iron” OR “Vitamin*” OR “Folate”.

All search results were imported into Covidence systematic review software for screening and selection of studies. The titles and abstracts were independently screened by two reviewers to identify any eligible studies. Full-text articles were then retrieved and evaluated by both reviewers using the predefined inclusion and exclusion criteria to confirm eligibility.

**Figure 1:**
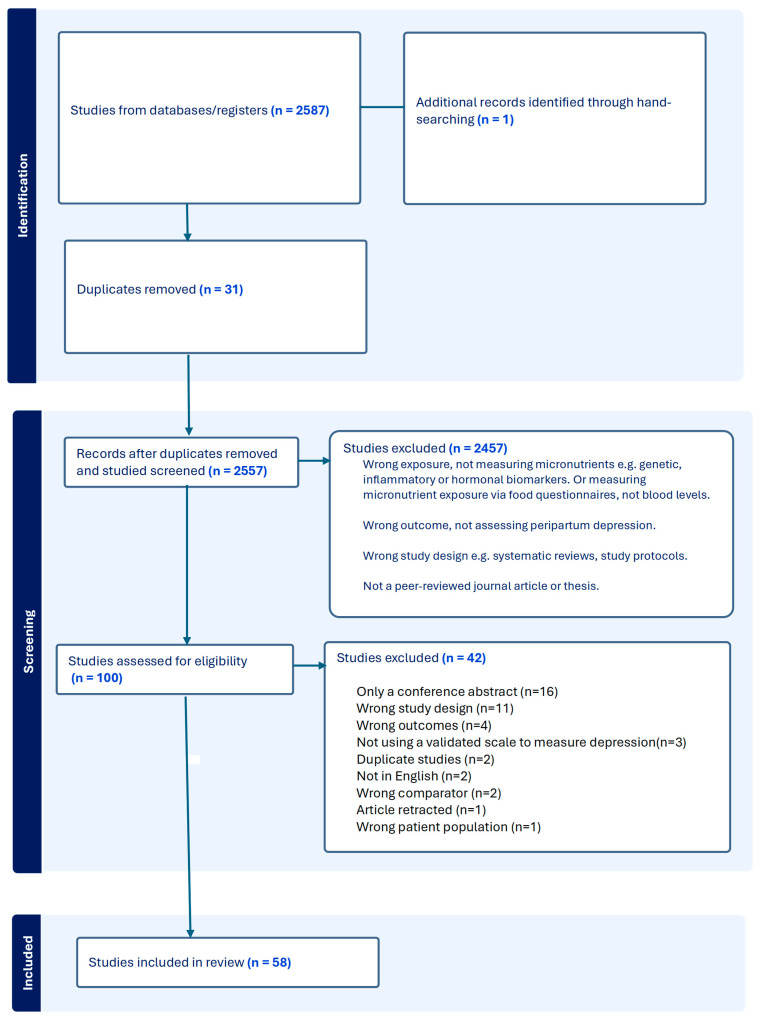
PRISMA diagram depicting the selection of studies for this review.

Any discrepancies in the assessment of study eligibility were resolved through a discussion involving an independent third reviewer. The full study selection process, which includes the number of articles screened, excluded, and included, is depicted in Figure 1.

### 2.2. Eligibility Criteria

The inclusion criteria for the studies included in this review are outlined below:

- Peer-reviewed journal articles or dissertations written in English.
- Population (P): Women of any age who were pregnant or within 12 months postpartum.
- Intervention/Exposure (I): Assessment of blood micronutrient levels, where micronutrients are defined as vitamins and minerals essential for human life [15]. Measurements in terms of serum levels, plasma levels, whole blood levels, or blood cell levels of the micronutrient were all considered acceptable.
- Comparator (C): The comparator groups include women with differing micronutrient levels, women without micronutrient deficiency, or no comparator group (as applicable to the study design).
- Outcome (O): Presence and/or severity of perinatal depression, determined either by a validated rating scale or by a clinical diagnosis according to recognised classification systems, such as the American Psychiatric Association’s Diagnostic and Statistical Manual of Mental Disorders (DSM) or the World Health Organization’s International Classification of Diseases (ICD) [16,17].

### 2.3. Quality Assessment

The quality of observational studies was assessed by a single reviewer using the Newcastle–Ottawa Scale (NOS) [18]. We acknowledge that independent dual assessment would have strengthened the rigour of this study, and this represents a limitation of the review [18]. Randomised controlled trials (RCTs) were assessed using the Joanna Briggs Institute (JBI) checklist [19]. The NOS evaluates a number of domains, including selection, comparability of groups, and ascertainment of outcome. Studies with scores of 0–3 are considered low quality, scores of 4–6 are considered moderate quality, and scores of 7–9 are considered high quality [20]. The JBI checklist is a descriptive measure of quality that explores domains of randomisation, concealment of allocation, blinding of allocation, and whether treatment groups were managed the same way outside of the intervention [19].

### 2.4. Statistical Analysis

A structured narrative synthesis was conducted to summarise and compare the findings across all included studies. Data was extracted from each article with regard to author and date of publication; conflicts of interest from authors; funding sources; study design; number of participants; the population from which participants were retrieved, including country and clinic setting; the micronutrients studied and the method that was undertaken; the timing of micronutrients tested in terms of the perinatal period, the depression rating tool used, and the cut-off; the timing of depression assessment in relation to the perinatal period; the statistical methods used to deduce associations between micronutrient levels and depression scores; and the measurement of confounding factors as well as the statistical analysis used to account for these. Descriptive statistics reported in the original publications, including medians, ranges, percentages of events, and confidence intervals, where available, were extracted and presented to outline the variability in clinical outcomes across studies and subgroups. Summary tables and figures were constructed to display study characteristics, intervention details, and reported outcomes in a clear and consistent format. Results were organised according to the micronutrient examined.

## 3. Results

### 3.1. Study Selection and Description of Studies

The search through six databases (PsycINFO, Web of Science, MEDLINE, Scopus, EMBASE, and CINHAHL) yielded 2587 records, including one dissertation. Hand-searching produced another relevant journal article. After 31 duplicates were excluded at this stage, 2556 records were screened by title and abstract. A total of 100 studies were selected for full-text evaluation, of which 58 met the eligibility criteria. Please see Figure 1, which depicts the selection of studies for this review in accordance with PRISMA guidelines.

### 3.2. Main Findings

Studies were grouped according to the type of micronutrient biomarker studied. The most studied micronutrient was vitamin D, with 28 studies examining its relationship with either prenatal or postnatal depression [21,22,23,24,25,26,27,28,29,30,31,32,33,34,35,36,37,38,39,40,41,42,43,44,45,46]. Regarding the other micronutrients, 13 articles studied iron [29,44,47,48,49,50,51,52,53,54], 12 articles studied folate [55,56,57,58,59,60,61,62,63,64], 10 articles studied vitamin B12 [59,60,62,63,64,65,66,67,68], 5 articles studied zinc [10,69,70,71], 3 studied magnesium [42,71,72], 3 studied copper [42,72,73], 2 studied selenium [72,74], 2 studied vitamin C [61,75], 1 studied calcium [42], 2 studied vitamin E [45,61], 1 studied vitamin A [45,61], 1 studied beta-carotene [61], 1 studied vitamin B2 [45], and 1 studied manganese [72]. A number of studies investigated more than one micronutrient [29,44,45,59,60,61,62,63,64,72]. Sample sizes varied from 31 participants (Ohsuga et al.) to 16,528 participants (Jani et al.) [40,53].

The Edinburgh Postnatal Depression Scale (EPDS) was the most common rating tool used in the studies to assess perinatal depression. In Australia, it is the recommended screening tool for depression in the perinatal period [11]. The EPDS is a 10-item validated self-rating scale developed by Cox et al. in 1987 [76] to screen for perinatal depression [16]. Depressive symptoms in most studies were assessed using the Edinburgh Postnatal Depression Scale (EPDS), with cut-off scores for probable depression varying between ≥10 and ≥13, which may influence reported prevalence and effect estimates. Other rating tools used to assess depression included the following: the Center for Epidemiologic Studies Depression Scale (CES-D); Beck Depression Inventory (BDI); Kessler Psychological Distress Scale (K-10); Depression Anxiety and Stress Scale (DASS-21); Patient Health Questionnaire (PHQ-9); Mini International Neuropsychiatric Interview (MINI); and Structured Clinical Interview for DSM (SCID). Table 1 summarises the findings of the review articles below.

#### 3.2.1. Vitamin D

Of the 28 studies examining vitamin D, 25 studies were observational in design and 3 were randomised controlled trials (RCTs). Six out of the eight studies examining depression scores in the antenatal period found a significant inverse relationship between vitamin D levels and depression scores in pregnancy [25,26,27,30,40,44]. The two largest studies in this review, Jani et al. (n = 16,528) and Brandenbarg et al. (n = 4101), supported this association. Jani et al., for example, found women with a high perinatal depression risk had increased odds of being vitamin D deficient (adjusted OR 1.321, 95% CI 1.105–1.579) [40]. Brandenbarg et al. demonstrated a significant inverse correlation between vitamin D status and antenatal depression scores (Spearman *p* = −0.188, *p* < 0.001) [25].

Nine studies exclusively examined the relationship between vitamin D and depression scores in the postnatal period [21,22,33,35,41,42,45,46,77]. Five studies demonstrated a significant inverse relationship between vitamin D levels and depression scores [21,22,33,46,77].

Eight studies that investigated the relationship between vitamin D levels and both antenatal and postnatal depression yielded mixed findings [23,29,31,32,34,37,38,41]. Lamb et al., for example, found a significant inverse relationship between vitamin D and EPDS scores antenatally and postnatally. Their study demonstrated that women with elevated depressive symptoms (i.e., EPDS score ≥ 10) had significantly lower vitamin D levels (t = −2.09, *p* = 0.039) [32].

However, Nassr et al. and Noshiro et al. did not find any significant association between vitamin D levels and antenatal or postnatal depression [34,41]. These null findings may reflect that studies with smaller sample sizes were not sufficiently powered to detect an association. Results from a study by King et al. suggested that women with vitamin D deficiency (defined as serum levels ≤ 20 ng/mL) had an odds ratio (OR) of 2.40 (95% CI 0.92–6.27) for elevated depression scores. However, this finding was not statistically significant (*p* = 0.07) [31].

While Accortt et al., 2021 did not find vitamin D to be significantly associated with perinatal depression risk, they found that a lower vitamin D metabolite ratio (VMR) was associated with postpartum depression (OR of 1.43 (95% CI 1.10–1.87, *p* = 0.007) [23]. Wang et al. found a significant relationship between vitamin D and postnatal depression, but not antenatal depression [37]. The authors found that for every 5 ng/mL increase in serum 25(OH)D, the EPDS score for ‘depressed mood’ decreased by 0.1 points [37].

In contrast, Williams et al. and Evanchuk et al. both found a significant relationship between vitamin D levels and antenatal depression scores, but not postnatal depression scores [29,38]. Williams et al. found that vitamin D at 12–20 weeks was a significant predictor of the BDI score at 12–20 weeks (*p* < 0.05) and at 34–36 weeks of gestation (*p* < 0.05) [38]. For every one unit increase in vitamin D in early pregnancy, there was an approximate 0.14 unit decrease in the antenatal BDI score (95% CI −0.26 to −0.017) [38]. Evanchuk et al. found that higher vitamin D levels were associated with lower EPDS scores in the third trimester (*p* = 0.001) but did not find any association between vitamin D levels and postpartum EPDS scores [29].

Three RCTs examined the effect of vitamin D supplementation on PPD [24,28,36]. None of these studies found a relationship between serum vitamin D levels and peripartum depression scores at baseline. Please see a summary of results in Table 1.

#### 3.2.2. Iron

A total of 13 studies assessed the association between iron status and iron-related biomarkers and perinatal depression. Of these, 12 were observational studies and 1 was an RCT. Five studies examined depression in the antenatal period, five examined postnatal depression, and two examined both time periods.

Only two studies found a significant association between deficiency of iron (or iron-related biomarkers) and antenatal depression. Dama et al. found the adjusted odds ratio for those who were iron-deficient in pregnancy developing depression was 2.51 (95% CI 1.14–5.52) [51]. The other study supporting a significant association was by Basutkar et al. (2022) [49]. This paper found a significantly higher mean EPDS score in the iron-deficiency anaemia (IDA) group (12.78 +/− 3.40) compared with the non-IDA group (8.82 +/− 3.12), 95% CI 2.94–4.87, *p* < 0.005 [49]. Ferritin concentration was one of the predictors of EPDS scores (correlation coefficient r = −0.50, *p* < 0.001) [49]. Serum iron was also negatively correlated with EPDS with a correlation coefficient, r = −0.38, *p* < 0.001 [49]. Pregnant individuals with iron deficiency were 12 times more likely to develop depression compared to those without iron deficiency [49]. The remaining three studies, by Hasdemir et al., Bodnar et al. and Basutkar et al. (2021), did not find any significant association between depression scores and iron deficiency [44,52,61].

Only one study (Albacar et al.) found a significant relationship between iron studies and depression scores in the postpartum period [47]. Ferritin concentrations were significantly lower in the group with postpartum depression (PPD) compared to the non-PPD group (15.4 +/− 12.7 mcg/L vs. 21.6 +/− 13.5 mcg/L, *p* = 0.002) [47]. Albacar et al. was the only study that measured inflammation as a covariate through CRP and found that ferritin persisted as a marker of postpartum depression even after CRP was taken into consideration through multivariate logistic regression analysis [47]. Both Ohsuga et al. and Evanchuk et al. examined the relationship between iron studies and depression scores during the antenatal and postnatal periods. Evanchuk et al. identified a significant relationship between low iron levels in mid-pregnancy and EPDS scores in the third trimester [29]. However, there was no relationship between serum ferritin levels and postpartum EPDS scores [29]. Ohsuga et al. found no significant difference in the median EPDS scores between individuals with non-anaemic iron deficiency (NAID) and those without [53]. However, within the NAID group, EPDS scores increased significantly from mid-pregnancy to one month postpartum [53]. Paoletti et al., in a randomised control trial, found no significant association between haematological or iron values at any timepoint and peripartum depression scores [54]. Please find a summary of these results in Table 2.

#### 3.2.3. Folate

Twelve studies examined the relationship between blood folate levels and perinatal depressive symptoms. Five of these studied the antenatal period, four studied the postnatal period, and three studied both time periods. Only two articles found a statistically significant relationship between folate levels and depressive symptoms. Chong et al. found a significant association between lower folate concentrations in those with probable antenatal depression compared to those without (mean +/− SSD: [27.3 +/− 113.8 vs. 40.4 +/− 336.5 nmol/L]; *p* = 0.011) [64]. However, they did not find any association between folate and postnatal depression [64]. The second study by Abou-Saleh et al. found lower folate levels in those with postpartum depression (*p* < 0.01) [60]. This study considered the effect of vitamin B12 on this association through stepwise multiple regression analysis; however, other covariate factors, such as inflammatory markers and supplementation, were not included. Three studies measured red cell folate, which is a more accurate reflection of folate status than serum folate [78], plasma folate, or dietary folate [78]. These three studies found no association between red blood cell folate levels and depression scores [56,62,68]. Please find a summary of these results in Table 3.

#### 3.2.4. Vitamin B12

There were 10 articles in total that examined the link between vitamin B12 and perinatal depression. Five articles examined the postnatal period only [57,59,60,66,67], three examined the antenatal period only [43,62,63], and two articles examined both time periods [64,65]. Three articles found a significant relationship between vitamin B12 levels and depressive symptoms in the postpartum period [60,66,67] and one in the antenatal period (Peppard et al.) [63]. Abou-Saleh et al. was the only study that showed a positive relationship between B12 levels and depressive symptoms; the other studies showed a negative relationship [60]. It is important to note that Abou Saleh et al.’s study had a number of limitations, including a relatively small sample size, a short follow-up period, and limited confounding factors considered, which may have resulted in an overestimation of the association [60]. Four of the ten studies into vitamin B12 examined the link between homocysteine levels and depression scores [59,62,65,67]. Only Aishwarya et al. found a significant association between homocysteine levels with postpartum depression. A positive association was found at both 24–28 h postpartum (*p* = 0.001) as well as six weeks postpartum (*p* = 0.001) [59]. Please find a summary of these results in Table 4.

#### 3.2.5. Zinc

All five articles studying serum zinc measured depressive symptoms in the postpartum period, and two studies also assessed the antenatal period. Four articles found an inverse association between zinc and depressive symptoms. Roomruangwong et al. found a significant inverse relationship between serum zinc and depressive symptoms both antenatally and postnatally using various assessment tools [10]. This was supported by Kavitha et al. [70], who found a strong inverse relationship (r = −0.24, *p* < 0.05) between zinc and postpartum depression. Wojcik et al. also found a negative association between serum zinc and EPDS scores in the postpartum period [71]. Indriasari et al., however, found the strength of the relationship between zinc and postpartum depression to be weak [79]. Only the Kurniati et al. study found no correlation [69]. Please find a summary of these results in Table 5.

#### 3.2.6. Copper

There were three studies that examined blood copper levels and perinatal depression [42,72,73]. In a retrospective cohort study of 902 patient records, of which 78 were women with postpartum depression, Crayton and Walsh found that women with a history of postpartum depression had significantly elevated serum copper levels (131 mcg/dL +/− 39 mcg/dL) compared to women with a history of depression but without postpartum depression (111 +/− 25 mcg/dL, *p* < 0.001) and to non-depressed controls (106 +/− 20 mcg/dL, *p* < 0.001) [73]. Bahramy et al. found that mean serum copper measured between 26- and 32-week gestation was significantly higher in women with depression compared to those without (*p* = 0.048) [42]. In contrast, Rokoff et al. found no association between erythrocyte copper concentrations and elevated EPDS at any timepoint [72]. Please find a summary of these results in Table 6.

#### 3.2.7. Other Micronutrients

No associations were found between peripartum depression and selenium [72,74], magnesium [42,71,72], B-carotene [61], vitamin C [47,75], vitamin A [61], and vitamin E [45,47]. Lin et al., however, did find plasma riboflavin level to be associated with decreased risk of postpartum depression (OR 0.747, 95% CI 0.566–0.987, *p* = 0.040) [45]. Please find a summary of these results in Table 7.

## 4. Methodological Quality

Only one study included in this review was found to be of low quality. Of the 54 observational studies, 8 studies were of moderate quality, and the remaining 45 studies were of high quality. The NOS scores and micronutrients studied in each of the observational studies are presented in Table 8. Table 9 presents the qualitative JBI quality assessment for the RCTs included in the review.

In general, most studies provided a reliable exposure measurement with clear descriptions of the method by which micronutrients were tested from the blood samples drawn. Due to the eligibility criteria, all studies used a standardised rating tool for assessment of depression symptoms, and only one article used a diagnosis based on a validated diagnostic standard, i.e., DSM-IV [73]. In a number of studies, it was not clear what cut-off score the authors used to define probable perinatal depression [57,68,69].

Most studies clearly described the source of the study populations. Sample sizes were, in general, of moderate to large size, with 43 out of 58 studies having sample sizes greater than 100. However, only 15 studies published a power calculation regarding their sample size [24,32,42,46,49,50,54,56,59,61,66,70,75]. Most of these had a power of at least 80% or over, with a significance level of 0.05 to detect a moderate effect size. Several studies fell short of the required number of participants needed to reach a power of 80% to detect a moderate effect size [30,72,75].

The majority of studies took into consideration multiple confounding factors and used appropriate statistical methods to account for their effects. These included regression-based models such as linear regression models, multiple logistic regression, and multivariate general linear model analysis, as well as stratified models. Three papers provided no clear description of any confounding factors [41,68,71]. The remaining 11 studies either took into account only a few confounding factors, were not clear on how the confounding factors were adjusted for statistically, or could have used more sophisticated statistical methods to adjust for these [28,42,53,54,57,59,60,69,70,73,74,75,79].

Multiple confounding factors were found to have a statistically significant association with depression scores. A number of articles supported an association between younger age and depression scores [25,37,64,70,72]. Repeatedly, an association was found between obesity and depression scores [25,27,40,43,55,63]. A number of studies suggested that lower education levels in the patients were positively associated with depression scores [25,33,55,64]. This contrasted with other studies, which reported that higher education levels were associated with depression scores [72]. A number of studies from India suggested that the middle socioeconomic group was actually associated with higher rates of perinatal depression [46,59,67]. The reason for this is unclear, but it may be due to greater access for the middle—upper class to healthcare, including psychiatric services. Studies were consistent in finding a positive association between unemployment and depression scores [46,47]. Ethnicity featured as an important confounding factor in some studies, with a recurrent finding of higher depression scores in Hispanic and African American patients compared to Caucasian patients [23,33,72]. Marital factors associated with higher depression scores included marital dissatisfaction [46,67,77], divorce or separation [27,33], polygamy [43], and consanguinity [49]. Studies from India suggested that dissatisfaction with the gender of the child was associated with depression scores [46,67]. A history of depression or mental illness was also found to have a strong relationship with depression scores [27,56,77]. For example, Blunden et al. found a history of a mental health condition had a relative risk of 1.80 (95% CI 1.50–2.15) *p* < 0.001 with postpartum depression [56]. However, only eleven articles mentioned a past history of mental illness as a confounding factor. There were many other confounding factors that associated positively with depression scores. Some of these included smoking [37], passive smoke exposure [43,64], drinking alcohol [37], formula feeding [33], pregnancy complications [59,62], mode of delivery [67], stressful life events [43,77], and unplanned pregnancy [67]. Breastfeeding [56] and a healthy diet [43] appeared to be protective factors against depression.

## 5. Discussion

This systematic review summarises the evidence of associations between a broad number of micronutrients and perinatal depression. The evidence was strongest for vitamin D, with 19 high-quality studies linking low vitamin D levels to perinatal depression. There was also evidence for an inverse association between iron studies, vitamin B12, and zinc levels and perinatal depression. There was less evidence for an association between folate levels and perinatal depression. There was little evidence for a relationship between other micronutrients and perinatal depression. Conversely, high serum copper levels were associated with depression.

Most of the studies included in this review were of high quality. Of the 58 studies included in this review, 31 studies supported a significant association between micronutrient level and antenatal or postnatal depression, and 27 studies found no association. Most of the studies that found a significant association suggested an inverse relationship between the micronutrient level and perinatal depression, i.e., that a micronutrient deficiency was associated with perinatal depression.

Copper is the only exception to this trend, with higher levels being associated with perinatal depression in two studies, albeit one of these studies was of poor quality [42,73]. Both serum copper and ceruloplasmin increase under the influence of inflammatory conditions. This was not considered as a covariate in the articles included [80]. Serum copper rises in pregnancy, likely as a result of rising oestrogen, and falls significantly in the postpartum reaching pre-pregnancy levels after six weeks [81]. Thus, the timing of copper testing in relation to the timing in the perinatal period is likely to affect copper levels also. Current literature suggests that an elevated cellular copper can lead to neuronal injury and induce oxidative stress and pro-inflammatory responses, potentially contributing to the development of depression [82]. Ni et al.’s systematic review of 21 observational studies supports this, with a finding that blood copper levels were higher in patients with depression [83]. This finding was also supported by Hulsbosch et al.’s study of 2036 pregnant women, which found that the copper-to-zinc ratio (Cu:Zn ratio) was independently associated with a persistently high negative affect, as measured by the Tilbury Distress Scale negative affect subscale (adjusted OR 1.52, 95% CI 1.13–2.04) [84].

In this review, the micronutrient with the greatest evidence base for its association with perinatal depression was vitamin D. All except one of the articles studying vitamin D were of high quality. Vitamin D studies accounted for 28 out of the 58 studies in this review. Of these studies, 19 supported a significant inverse association between vitamin D and perinatal depression. Eight studies found no association at all. The variation in findings may be for a number of reasons. The null findings from these studies may reflect studies with smaller sample sizes that were not sufficiently powered to detect an association. Secondly, it may be difficult to observe an association in populations with a high prevalence of vitamin D deficiency, as both groups of women have high rates of deficiency [34]. Sunlight exposure and seasonality are important confounding factors affecting vitamin D levels. However, only four studies attempted to measure sunlight exposure as a confounding factor [21,36,44,46], and only ten studies measured seasonality [21,26,27,28,30,32,35,37,39,41,77]. The existence of 11 systematic reviews on vitamin D and perinatal depression is notable, as no other micronutrient has been similarly reviewed [85,86,87,88,89,90,91,92,93,94,95]. In general, these reviews supported an inverse relationship between vitamin D and perinatal depression scores.

The strong evidence base for vitamin D may stem from the ease, reliability, and low cost of testing vitamin D serum levels, making it relatively easy to research. Vitamin D is unique in that it is also a neuro-steroid [96,97]. Various biological mechanisms have been hypothesised in the literature to explain the association between low vitamin D levels and depression. These include the role of vitamin D in the synthesis of neurotransmitters such as acetylcholine, dopamine, serotonin, and gamma aminobutyric acid; its antioxidant and anti-inflammatory effects in the central nervous system; its regulation of neuronal levels of calcium; and its role in enhancing nerve growth factors, such as BDNF [98,99,100,101,102]. Furthermore, vitamin D acts as a transcriptional regulator for many genes and also has roles in neuronal function and plasticity [98].

The second largest number of articles were regarding iron and its association with perinatal depression. Five out of twelve studies on iron suggested a significant inverse relationship with perinatal depression, i.e., iron deficiency was associated with higher depression scores. This relationship with postnatal depression is biologically plausible as iron is an essential co-factor in the synthesis of neurotransmitters such as serotonin, melatonin, norepinephrine, and dopamine, as well as myelin synthesis in the central nervous system [103]. Iron also plays key roles in the functioning of the hippocampus as well as the pre-frontal cortex, which are important brain networks involved in the pathophysiology of major depressive disorder [103]. In animal models, iron deficiency during the perinatal period in mice was associated with impaired maternal care postpartum as well as impaired neurodevelopment in the infant mice [103]. However, one explanation for the mixed outcome from these high-quality studies may be due to the fact that iron status in the body is measured through multiple biomarkers (serum ferritin, serum iron, and transferrin saturation). This complicates the interpretation of the relationship of iron with depression scores and make direct associations more difficult to establish. Moreover, ferritin is an acute-phase reactant that can mask iron deficiency when it rises with inflammation [104]. Only one study in this review considered inflammation as a covariate [47]. Studies also varied in whether they focused on anaemic or non-anaemic patients, which may represent an important confounding factor influencing the observed relationships with depression outcomes. 

Folate and vitamin B12 play a central role in the DNA methylation process, which is crucial to the normal development and function of the central nervous system [105]. There is substantial evidence to support that inappropriate methylation patterns can contribute to a range of neuropsychiatric issues such as depression, autism, and schizophrenia [105]. Deficiencies in these micronutrients can result in elevated homocysteine levels, which have been associated with depression [8,106] and also cognitive impairment [107,108]. Deficiencies in vitamin B12 and folate can become overt during pregnancy and lactation, when demands of the growing foetus and delayed repletion impose significant nutritional demand [7,109]. In particular, it is well known that deficiencies of folate in pregnancy can result in foetal neural tube defects [109].

This review found limited evidence for the association between vitamin B12 and perinatal depression despite these studies being of high quality. Three articles suggested an inverse relationship, and one study (Abou-Saleh et al.) suggested a positive relationship with depression scores [60]. It is important to note that the Abou-Saleh et al. study had a number of limitations, as described previously. 

Similarly, regarding folate, while the majority of studies examining this micronutrient were of high quality, only two of the twelve studies found a significant association between blood folate levels and depression scores [60,64]. For folate, the absence of a significant association in some studies may reflect an incomplete adjustment for other one-carbon metabolites, such as vitamin B12 and homocysteine, which are biochemically interdependent and may confound the relationship between folate status and perinatal depression.

There were five studies that studied blood zinc levels and perinatal depression, of which three demonstrated a strong inverse relationship. However, the quality of these studies was poorer when compared with the studies of the other micronutrients. Kurniati et al. did not provide clear cut-off scores to define depression, and it was unclear at what timepoint postpartum participants were recruited [79]. Wojcik et al. did not clearly identify any confounding factors and did not directly statistically quantify the nature and strength of the association between serum zinc and depression scores [71]. Only Roomruangwon et al. appeared to consider the diurnal variation of zinc and measured fasting morning zinc levels [10]. Roomruangwong et al. was also the only study that considered CRP as a covariate, which is important considering that zinc levels decrease in inflammatory states [110]. Roomruangwong et al. found there was a significant inverse relationship between zinc and CRP in the prenatal period. Both lower zinc and higher CRP were associated with prenatal and postnatal depression [10].

With regard to the other micronutrients measured, the evidence base was very limited. In general, no significant association was found between these micronutrient levels and perinatal depression, except for a positive association between riboflavin and depression scores [45]. Further research is required to support an association between any of these micronutrients and perinatal depression. 

This systematic review found a significant link between low vitamin D levels and perinatal depression. This review provides evidence that antenatally screening routinely for vitamin D enables supplementation and potentially reduces the risk of peripartum depression. Vitamin D supplementation in perinatal women with a deficiency is likely to have multiple health benefits for the mother and baby. Severe vitamin D deficiency can lead to delayed early infant motor development as well as growth retardation, rickets, osteomalacia, and hypocalcaemia in children, and it has a critical role in bone mineralisation [111,112]. There is also emerging evidence that vitamin D deficiency may be related to neurodevelopmental disorders [113,114,115] as well as allergic disorders in children [116,117,118] because it has important roles in brain development and functioning [96,119] and in the regulation of the immune system [120]. In adult women, it is well known that vitamin D deficiency is associated with the development of osteoporosis [121]. However, there is also emerging evidence for the association between low vitamin D and a number of other conditions, including autoimmune conditions, cancers, and cardiovascular disease [120].

The risks of developing vitamin D deficiency may be higher in postpartum women who may struggle to find the time to obtain sufficient sunlight exposure, and even higher in women with postpartum depression who often struggle to leave the home. Women of culturally diverse backgrounds are also likely to be at a higher risk of vitamin D deficiency due to decreased skin absorption for those with darker skin pigmentation and also cultural practices related to veiling [122]. Therefore, routine antenatal screening for vitamin D deficiency and appropriate supplementation offers an opportunity not only to potentially prevent perinatal depression but also to promote broader maternal and infant health.

In Australia, vitamin D levels are not routinely tested antenatally. The current Royal Australian and New Zealand guidelines for Obstetrics and Gynaecology state, with regard to vitamin D testing, not to “test Vitamin D levels in pregnancy as part of routine pregnancy screening, regardless of maternal risk factors” [123]. Medicare currently will not subsidise routine testing for vitamin D in the perinatal period [124]. Thus, it is highly likely that vitamin D deficiency in pregnancy may be underdiagnosed in the Australian perinatal population, and a valuable opportunity to reduce the risk of perinatal depression through vitamin D supplementation is being overlooked.

### Strengths, Limitations, and Future Directions

This systematic review has a number of strengths in that it involved a comprehensive search of the literature that spanned multiple databases and involved forward citation-tracking of articles. It aimed to include a detailed assessment of the studies’ methodological quality. However, there were several limitations of this review. There was significant variability across the studies with regard to the micronutrients measured, the instruments used to assess depressive symptoms, and the cut-offs used to define probable depression. For example, there were differences in the EPDS thresholds applied across studies (e.g., ≥10 vs. ≥13), which likely contributed to the variability in reported associations between micronutrient levels and perinatal depression. Variability in findings across studies may reflect differences in dietary patterns, baseline nutritional status, and genetic polymorphisms such as MTHFR that influence folate and B12 metabolism. Another explanation for this variability is that articles studied women from various geographical locations, ethnicities, and cultures. Confounding factors such as seasonality, inflammation, body mass index, socioeconomic status, and comorbidities may also partly explain the inconsistent results. In addition, methodological variability, including differences in assay techniques, diagnostic tools for depression, and definitions of micronutrient deficiency, likely contributes to heterogeneity. Variation in the timing of micronutrient assessment across studies—such as measurement in early versus late pregnancy or postpartum—may also contribute to inconsistent findings. Micronutrient levels fluctuate throughout pregnancy and after delivery due to physiological changes, haemodilution, dietary intake, and supplement use, which may affect their observed relationship with depressive symptoms. Micronutrient deficiencies may be more difficult to detect in well-nourished individuals from higher socioeconomic populations compared with lower socioeconomic areas, which may have a greater prevalence of malnutrition [61]. As a result of this variability, a meta-analysis or formal pooled analysis of these results was not possible.

Due to the variability of association measures across the papers, a meta-analysis could not be provided. It is possible that publication bias may be present, as studies finding an association may be more likely to be published than those not finding an association. The scope of this review was limited to measures of micronutrient levels in blood. However, the accuracy, availability, and cost of blood sampling micronutrient levels vary. For some micronutrients, assessment of dietary patterns or urinary micronutrient concentrations may be more accurate than blood testing.

## 6. Conclusions

This systematic review found a significant association between low vitamin D levels and perinatal depression. Currently, the Royal Australian and New Zealand College for Obstetricians and Gynaecologists (RANZCOG) guidelines do not recommend the routine screening of vitamin D levels antenatally [123]. This review provides evidence for routinely screening for vitamin D antenatally to enable supplementation and potentially reduce the risk of perinatal depression. There is mixed and conflicting evidence regarding other micronutrients, including iron, folate, vitamin B12, zinc, and copper, and thus further research is needed. Variability in findings across studies likely reflects differences in dietary patterns, baseline nutritional status, genetic polymorphisms, and variability in the timing of measuring the micronutrient. Differences in the cut-off thresholds applied to depression assessment tools (such as the EPDS) are likely to contribute to variability in the associations between micronutrients and perinatal depression. Confounding factors such as seasonality, inflammation, body mass index, socioeconomic status, and comorbidities may partly explain the inconsistent results. Future research needs to be adequately powered, use appropriate statistical methods to account for multiple confounding factors, and provide clear cut-off scores for defining cases of perinatal depression. Such research may help clarify the role of micronutrients in both the prevention and adjunctive treatment of perinatal depression. In addition, future studies could incorporate precision-nutrition approaches to account for individual variability in nutrient metabolism, genetic polymorphisms, and dietary intake. In addition, Mendelian randomization studies may help clarify whether observed associations between micronutrient status and perinatal depression reflect causal relationships or residual confounding. Future research into this area may help inform the role of micronutrients in the prevention of perinatal depression and as adjunctive treatments for perinatal depression.

## Figures and Tables

**Table 1:** Summary of findings for vitamin D and perinatal depression.

Author	Study Type	Marker and Method Assay	(N)	Time of Testing	Ax* Tool	Timing of Ax*	Results
Brandenbarg et al. [25]	CRS	25[OH]D via enzyme immunoassay	4101	Median: 13 wks GA	CES-D ≥ 16	14–18 wks GA	↓ Vit D → ↑ EPDS (Spearman *p* = −0.188 (*p* < 0.001) Adj. OR for vit D def. and EPDS scores: 1.48 (95% CI 1.13–1.95)
Cassidy-Bushrow et al. [26]	CRS	25[OH]D via chemiluminescence immunoassay	178	Median: 9.5 wks GA	CES-D ≥ 16	14–18 wks GA	Sig. inverse assoc. btw log vit D and CES-D ≥ 16 (OR = 0.54, 95% CI 0.29–0.99, *p* = 0.046)
Cunha Figueiredo et al. [27]	CHT	25[OH]D and 1,25[OH]_2_D via LC-MS	128	5–13 wks GA; 20–26 wks GA; 30–36 wks GA	EPDS ≥ 13	Assessed at each trimester of pregnancy	↑ Vit D → ↓ EPDS One-unit ↑ in vit D conc → 2% ↓ in the odds of AND* (Adj. OR = 0.98, 95% CI 0.96–0.99, *p*-value 0.047).
Huang et al. [30]	CRS	25[OH]D_2_ and 25[OH]D_3_ via LC-MS	498	15 wks GA	DASS-21 (≥14 mod dep^n^); PHQ-9 (≥10 mod dep^n^)	Mean: 15 wks GA	↓ Vit D → ↑ DASS-21. Lowest quartile of Vit D conc had 2.6 point ↑ DASS-21 scores cf highest quartile.
Woo et al. [39]	CHT	25[OH]D via radioimmunoassay	125	24–32 wks GA	EPDS ≥ 12	24–32 wks GA	No assoc.
Accortt et al., 2021 [23]	CHT	25[OH]D via chemiluminescence immunoassay	89	28–30 wks GA	BDI and CES-D ≥ 16	BDI at 28–30 wks GA; CES-D at 6–10 wks PP	PPD group had lower vit D metabolite ratio (VMR). A lower VMR had an OR for PPD of 1.43 (1.10–1.87), *p* = 0.007.
King et al. [31]	CHT	25[OH]D via radioimmunoassay	105	8–12 wks GA; 24–28 wks GA; 6–8 wks PP; 10–12 wks PP	EPDS ≥ 10	8–12 wks GA; 24–28 wks GA; 6–8 wks PP; 10–12 wks PP	Adj. OR for vit D def. and dep^n^ scores was 2.40 (95% CI 0.92–6.27) but not statistically sig. (*p* = 0.07)
Lamb et al. [32]	CHT	25[OH]D via LC-MS	88	14 wks GA; 32 wks GA; 10 wks PP	EPDS ≥ 10	14 wks GA; 32 wks GA; 10 wks PP	↑ EPDS → ↓ Vit D at all three timepoints (t = −2.09, *p* = 0.039)
Nassr et al. [34]	CHT	25[OH]D via electro-chemiluminescence	80	Third trimester	EPDS (Arabic) ≥ 13	Third trimester of pregnancy; 6 mths PP	No assoc.
Vaziri et al. [36]	RCT	25[OH]D via chemiluminescence immunoassay	169	26–28 wks GA and 48 h PP	EPDS (Persian) ≥ 9	26–28 wks GA; 38–40 wks GA; 4 wks PP and 8 wks PP	dep^n^ scores ↓ in vit D grp cf. control grp at 38–40 wks GA (*p* = 0.01) and 4–8 wks PP (*p* < 0.001).
Wang et al. [37]	CRS	25[OH]D_2_ and 25[OH]D_3_ to give total of 25[OH]D.	1805	Antenatal and postpartum (at time of enrolment)	EPDS (Chinese) ≥ 13	Antenatal and postpartum (at time of enrolment)	No assoc. antenatally Vit D def. → ↑ PPD (OR = 1.71, 95% CI 1.01–2.88, *p* = 0.044).
Williams et al. [38]	RCHT	25[OH]D via radioimmunoassay	105	12–20 wks GA; 34–36 wks	EPDS (≥9), BDI and MINI	12–20 wks GA; 26–28 wks GA; 34–36 wks GA and 6–8 wks PP.	↑ Vit D → ↓ BDI antenatally (*p* < 0.05) For each one-unit ↑ Vit D → ↓ 0.14 point BDI at 12–20-wks (95% CI −0.26 to −0.017). Nil assoc. postnatally.
Dabbaghmanesh et al. [28]	RCT	25[OH]D—method not clear	98	26–28 wks GA and 4 wks PP	EPDS ≥ 13	26–28 wks GA and 4 wks PP	↓ Mean depression scores in the vit D suppl. grp at 4 wks PP (4.48 +/− 3.30) cf. control grp (7.07 +/− 4.52).
Murphy et al. [33]	CHT	25[OH]D—rapid radioimmunoassay	978	4–6 wks PP and monthly thereafter until 7 mths PP	EPDS ≥ 9	4–6 wks PP and monthly thereafter until 7 mths PP	↓ Vit D → ↑ EPDS scores over time (*p* = 0.02).
Uslu Yuvaci et al. [35]	CRS	25[OH]D_3_—chemi-luminescence immunoassay	75	4–6 wks PP	EPDS ≥ 13	4–6 wks PP	No assoc.
Abedi et al. [21]	CC	25[OH]D via ELIZA	120	6–8 wks PP	BDI, cut-off score unclear	4–6 wks PP	Vit D sig. ↓ in women with PPD grp cf. control grp, *p* = 0.001. Vit D < 20 ng/mL were more likely to have PPD, OR 3.30 (95% CI 1.32–8.24), *p* value = 0.01.
Accortt et al., 2016 [22]	CHT	25[OH]D—via chemi-luminescence	91	First trimester	EPDS ≥ 12	4–6 wks PP	↑ Log vit D → ↓ EPDS scores (adjusted β = −0.209, *p* = 0.058) inflammatory markers moderated the effect (*p* < 0.05)
Pillai et al. [46]	CC	25[OH]D via ELISA	660	6 wks PP	EPDS (Tamil or English) ≥ 12	6 wks PP	Negative correlation btw vit D levels and EPDS scores (r = −0.19, *p* < 0.001). PPD grp had greater Vit D def. (35%) and insufficiency (43%) cf control grp (29% and 35%, respectively).
Amini et al. [24]	RCT	25[OH]D via ELISA	76	1–6 mths PP	EPDS ≥ 12	1–6 mths PP	Greatest improvement in EPDS was in the vit D suppl + Ca placebo grp. Vit D increased from 39.82 to 58.03, (*p* value < 0.001). PPD scores fell by 4.16 points, *p* = 0.004. No sig. change in EPDS scores in the placebo grp.
Jani et al. [40]	CHT	25[OH]D—method not clear	16,528	14 wks GA	EPDS ≥ 13	12–14 wks GA	↑ Depression scores had ↑ odds of being vitamin D def. Adjusted OR 1.321, 95% CI 1.105–1.579
Fu et al. [77]	CHT	25[OH]D—via electro-chemiluminescence	213	24–48 h PP	EPDS (Chinese) ≥ 12	3 mths PP	Vit D was a predictor of PPD with adjusted OR of 0.81 (95% CI 0.70–0.92, *p* < 0.0001) Vit D levels sig. in PPD cf. those without (14.3 vs. 8.3, *p* < 0.0001)
Noshiro et al. [41]	CHT	25[OH]D (from 25[OH]D_2_ + 25[OH]D_3_) via electro-chemiluminescence	99	24–27 wks GA, 33–35 wks GA and 1 mth PP	EPDS ≥ 9	3 days PP and 1 mth PP	No assoc.
Bahramy et al. [42]	CRS	25[OH]D via ELISA	200	26–32 wks GA	EPDS (≥13), DASS-21	26–32 wks GA	No assoc.
Lin et al. [45]	CRS	25[OH]D via electro-chemiluminescence	120	6–8 wks PP	EPDS (Chinese) ≥ 10	6–8 wks PP	No assoc.
Basutkar et al. [44]	CRS	25[OH]D—method not clear	120	26–28 wks GA	EPDS (Tamil) ≥ 8	26–28 wks PP	↑ Vit D → ↓ EPDS (f = −0.294, *p* = 0.001) E very one unit ↑ vit D → ↓ EPDS by 0.236 (95% CI −0.377 to −0.96, *p* = 0.01)
Evanchuk et al. [29]	CHT	25[OH]D_3_ + epi-25[OH]D_3_ via LC-MS	627	Each trimester of pregnancy and 3 mths PP	EPDS ≥ 13	3 mths PP	↑ Vit D → ↓ EPDS in third trimester (*p* = 0.001).
Al-Sabah et al. [43]	CRS	25[OH]D via electro-chemiluminescence	1070	2nd or 3rd trimester	EPDS (Arabic) ≥ 13	2nd or 3rd trimester	No assoc.
Bodnar et al. [61]	CRS	25[OH]D via ELISA	135	20 wks GA	SCID (DSM-IV)	20-, 30- and 36 wks GA	No assoc.

Abbreviations for tables: N = participant number; Ax*: assessment; CRS: Cross-sectional; CHT: Cohort; CC: Case–control; RCT: Randomised controlled study; RCHT: Retrospective cohort study; Se: selenium; Cu: copper; Mn: manganese; Mg: magnesium; Ca: calcium; Zn: zinc; Vit D: Vitamin D; 25[OH]D: serum-25-hydroxy vitamin D; LC-MS: liquid chromatography mass spectrometry; ELISA: Enzyme-linked immunosorbent assay; Fe: iron; Vit B12: Vitamin B12; GA: gestational age; wks: weeks; mths: months; AND*: antenatal depression; PPD: postpartum depression; conc: concentration; def.: deficiency; btw: between; PP: postpartum; grp: group; sig: significant; cf. compared with; suppl.: supplementation; assoc.: association; adj: adjusted, dep^n^: depression; EPDS: Edinburgh Postnatal Depression Score; CES-D: Centre for Epidemiological studies; and K-10: Kessler Psychological Distress.; ↓: decreased; ↑: increased; →: associated with.

**Table 2:** Summary of findings for iron studies and perinatal depression.

Author	Study Type	Markers Used	(N)	Time of Testing	Ax* Tool	Timing of Ax*	Results
Hasdemir et al. [52]	CRS	FBC, serum iron, TIBC, t/f sat, ferritin	408	24 wks GA	EPDS ≥ 12	~24 wks GA	No assoc.
Ohsuga et al. [53]	CRS	Hb, MCV, MCH, MCHC, ferritin	31	<16 wks GA; 24–34 wks GA; 35 wks GA	EPDS ≥ 8	Mid-pregnancy and 1 mth PP	No assoc.
Albacar et al. [47]	CHT	Serum iron, t/f sat, ferritin, CRP	729	48 h PP	EPDS (Spanish) ≥ 9	48 h PP; 8 wks PP and 32 wks PP	PPD group had ↓ ferritin levels cf. non-PPD group (*p* = 0.002)
Armony-Sivan et al. [48]	CHT	FBC, Hb, MCV, ferritin, sTfR*	567	Mid-pregnancy and late pregnancy	EPDS (Chinese) ≥ 10	6 wks PP	No assoc.
Chandrasekaran et al. [50]	CRS	Hb, StfR, ferritin	103	24 h PP and 3 wks PP	EPDS ≥ 10	24 h PP; 3 wks PP and 6 wks PP	No assoc.
Paoletti et al. [54]	RCT	Hb, serum iron, ferritin	852	Day 3 PP; Day 15 PP; Day 30 PP	EPDS ≥ 12	Day 3 PP; Day 15 PP and day 30 PP	No assoc.
Lin et al. [45]	CRS	FBC, ferritin	120	6–8 wks PP	EPDS (Chinese) ≥ 10	6–8 wks PP	No assoc.
Basutkar et al., 2021 [44]	CRS	Hb, ferritin	120	26–28 wks GA	EPDS (Tamil) ≥ 8	26–28 wks PP	No assoc.
Evanchuk et al. [29]	CHT	Ferritin, sTfR, Hepcidin	627	Each trimester and 3 mths PP	EPDS ≥ 13	3 mths PP	↓ Serum ferritin mid-pregnancy → ↑ EPDS scores in third trimester (β: −0.8; 95% CI −1.5, −0.01) No assoc. for Fe postpartum.
Basutkar et al., 2022 [49]	CRS	FBC (inc Hb, MCV, MCH, MCHC), Hct, serum iron, ferritin	210	2nd trimester	EPDS ≥ 14	13–28 wks GA	↓ Fe markers → ↑ EPDS scores 1. Ferritin: r = −0.50, *p* < 0.001; 2. Serum iron: r = −0.038, *p* < 0.001
Dama et al. [51]	CRS	Ferritin	142	≥20 wks GA	EPDS ≥ 12	20wks GA	↓ Ferritin → ↑ Depression scores Adj OR for dep^n^ in iron def. was 2.51 (95% CI 1.14–5.52).
Bodnar et al. [61]	CRS	Ferritin	135	20 wks GA	SCID (DSM-IV)	20-, 30- and 36 wks GA	No assoc.
Noshiro et al. [41]	CHT	Ferritin, iron, TIBC	99	24–27 wks GA, 33–35 wks GA and 1 mth PP	EPDS ≥ 9	3 days PP and 1 mth PP	No assoc.

Abbreviations: FBC: full blood count; Hb: haemoglobin; TIBC: total iron binding capacity; MCV: mean cell volume; MCH: mean cell haemoglobin; sTfR*: soluble transferrin receptor; Hct: haemocrit; Fe: iron; and CRP: C-reactive protein; Ax*: assessment.

**Table 3:** Summary of findings for folate and perinatal depression.

Author	Study Type	(N)	Timing of Testing	Depression Screening Tool, Cut-Off	Timing of Ax*	Main Outcome
Avalos et al. [55]	CRS	318	15 wks GA	CES-D ≥ 21	15 wks GA	No assoc.
Hasdemir et al. [52]	CRS	408	24 wks GA	EPDS ≥ 12	~24 ks GA	No assoc.
van Lee et al. [58]	CHT	1247	26–28 wks GA	EPDS ≥ 15 antenatal; EPDS ≥ 13 postnatal	26–28 wks GA and 3 mths PP	No assoc.
Morris et al., 2020 [68]	CRS	305	>15 wks GA	EPDS - cut-off score unclear	15 wks GA; 1–2 wks PP; 1–2 mths PP and 3–4 mths PP	No assoc.
Blunden et al. [56]	CRS	1976	~11 wks GA	EPDS ≥ 13	6 mths PP 12 mths PP	No assoc.
Lukose et al. [62]	CRS	365	11.5 wks GA	K-10 ≥ 6	~11.5 wks GA	No assoc.
Chong et al. [64]	CHT	709	26–28 wks GA	EPDS ≥ 15 antenatal; EPDS ≥ 13 postnatal	26–28 wks GA and 3 mths PP	↓ Folate → ↑ AND (*p* = 0.001) Antenatal: mean folate 27.3 +/− 113.8 in depressed group vs. 40.4 +/− 336.5 nmol/L in non-depressed group; *p* = 0.011
Abou-Saleh et al. [60]	CRS	62	Third trimester; Day 7 PP	EPDS (Arabic) ≥ 11	Day 7 PP	↓ Folate → PPD (*p* < 0.01)
Aishwarya et al. [59]	CC	103	24–48 h PP; 6 wks PP	EPDS ≥ 10	24–48 h PP and 6 wks PP	No assoc.
Peppard et al. [63]	CRS	174	Antenatally	PHQ-9 ≥ 10	Antenatally	No assoc.
Al-Sabah et al. [43]	CRS	1070	2nd or 3rd trimester	EPDS (Arabic) ≥ 13	2nd or 3rd trimester	No assoc.
Bodnar et al. [61]	CRS	135	20 wks GA	SCID (DSM-IV)	20-, 30- and 36 wks GA	No assoc.

Ax*: assessment.

**Table 4:** Summary of findings for vitamin B12 and perinatal depression.

Author	Study Type	(N)	Testing Timing	Ax* Tool	Ax* Timing	Results
Al-Sabah et al. [43]	CRS	1070	2nd or 3rd trimester	EPDS (Arabic) ≥ 13	2nd or 3rd trimester	Vit B12 → inverse assoc with EPDS (*p* = 0.009).
Lukose et al. [62]	CRS	365	11.5 wks GA	K-10 ≥ 6	~11.5 wks GA	No assoc.
Chong et al. [64]	CHT	709	26–28 wks GA	EPDS ≥ 15 antenatal; EPDS ≥ 13 postnatal	26–28 wks GA and 3 mths PP	No assoc.
Abou-Saleh et al. [60]	CRS	62	3rd trimester; Day 7 PP	EPDS (Arabic) ≥ 11	Day 7 PP	↑ Vit B12 → ↑ EPDS scores (r = 0.39, *p* < 0.01).
Aishwarya et al. [59]	CC	103	24–48 h PP; 6 wks PP	EPDS ≥ 10	24–48 h PP and 6 wks PP	No assoc.
Peppard et al. [63]	CRS	174	Antenatally	PHQ-9 ≥ 10	Antenatally	↓ Vit B12 → ↑ dep^n^ scores. OR = 3.82, 95% CI (1.10–13.31), *p* < 0.04.
Batalha et al. [65]	CHT	101	Third trimester; Day 2–8 PP; 28–50 days PP; 88–199 days PP	EPDS ≥ 11	Third trimester of pregnancy	No assoc.
Cruz-Rodriguez et al. [66]	CHT	336	12 wks GA; 36 wks GA	EPDS (Spanish) ≥ 10	~ 54 days PP	↑ Vit B12 → ↓ EPDS in 1st trimester (B = −1.267, CI −2.461 to −0.073, *p* = 0.038)
Dhiman et al. [67]	CC	434	6 wks PP	EPDS (Tamil) ≥ 10	6 wks PP	↓ Vit B12 → ↑ EPDS Lowest B12 quartile had 4.53 times likelihood of PPD (*p* = 0.001)
Morris et al., 2019 [57]	RCHT	365	1–2 wks; 1–2 mths PP; 3–4 mths PP	EPDS cut-off not specified	1–2 wks; 1–2 mths PP; 3–4 mths PP	No assoc.

Ax*: assessment.

**Table 5:** Summary of results for zinc and perinatal depression.

Author	Study Type	(N)	Testing Timing	Ax* Tool	Ax* Timing	Results
Roomruangwong et al. [10]	CRS	71	3rd trimester and 4–6 wks PP	MINI, EPDS (≥11) and BDI	3rd trimester, 4–6 wks PP	Zn inversely assoc. with dep^n^ scores antenatally and postnatally. EPDS (r = −0.425, *p* < 0.001, n + 71), HAMD (r = −0.478, *p* < 0.001, n = 71) and BDI (r = −0.507, *p* < 0.001, n = 71).
Kurniati et al. [69]	CRS	70	PP unclear timepoint	EPDS, unclear cut-off score	PP unclear timepoint	No assoc.
Indriasari et al. [79]	CRS	87	≥4 wks PP	EPDS ≥ 13	4 wks PP	Inverse assoc. btw Zn and EPDS (r = −0.063, *p* = 0.564)
Kavitha et al. [70]	CC	80	Up to 6 mths PP	EPDS ≥ 10	Up to 6 mths PP	Inverse assoc. btw Zn and EPDS (r = −0.24, *p* < 0.05)
Wojcik et al. [71]	CHT	58	3rd trimester; 3rd day PP; 30th day PP	EPDS ≥ 9, BDI	BDI at 3rd trimester, EPDS 3rd and 30th day PP	Inverse relationship found btw Zn and dep^n^ scores but not qualified statistically. Day 3: 42% with PPD—mean Zn 0.61 mg/mL (+/−0.01). Day 30: 29 % with PPD—mean Zn 0.80 mg/L (+/−0.02).

Ax*: assessment.

**Table 6:** Summary of results for copper and perinatal depression.

Author	Study Type	(N)	Testing Timing	Ax* Tool	Ax* Timing	Results
Crayton et al. [73]	RCHT	902	Unspecified	DSM-IV based Dx	Unspecified	↑ Cu is associated with PPD. Mean Cu level 131 mcg/dL in PPD cf 111 mcg/dL +/− 25 for women without PPD, *p* < 0.001).
Bahramy et al. [42]	CRS	200	26–32 wks GA	EPDS (cut-off score ≥ 13), DASS-21	26–32 wks GA	↑ Mean serum Cu in AND cf those without (100.6 vs. 93.0, *p* = 0.048). No other associations were found.
Rokoff et al. [72]	CRS	1226	Median 9.6 wks GA	EPDS ≥ 13	Mid-pregnancy, 6 mths PP, 12 mths PP	No assoc.

Ax*: assessment.

**Table 7:** Summary of findings of other micronutrients and perinatal depression.

Author	Mn* Studied	Study Type	(N)	Testing Timing	Ax* Tool	Ax* Timing	Results
Rokoff et al. [72]	Cu, Mg, Mn, Se, Zn	CRS	1226	Median 9.6 wks GA	EPDS ≥ 13	Mid-pregnancy, 6 mths PP, 12 mths PP	No associations found
Jin et al. [74]	Se	CRS	87	3 mths PP; 6 mths PP; 12 mths PP	EPDS ≥ 10	3 mths PP; 6 mths PP; 12 mths PP	No associations found
Bahramy et al. [42]	Vitamin D, Ca, Mg, Cu	CRS	200	26–32 wks GA	EPDS ≥ 13, DASS-21	26–32 wks GA	↑ Mean serum Cu in depression cf those without (100.6 vs. 93.0, *p* = 0.048). No other associations were found.
Wojcik et al. [71]	Zn, Mg	CHT	58	3rd trimester; 3rd day PP; 30th day PP	EPDS ≥ 9, BDI	BDI at 3rd trimester, EPDS 3rd and 30th day PP	No assoc. found for Mg.
Bodnar et al. [61]	Folate, Vitamins A, C, D, E, B-carotene	CRS	135	20 wks GA	SCID (DSM-IV)	20-, 30- and 36 wks GA	No associations found
Lin et al. [45]	Iron studies, Vitamins B2, D and E	CRS	120	6–8 wks PP	EPDS (Chinese) ≥ 10	6–8 wks PP	↑ Riboflavin → ↓ PPD. OR = 0.747, 95% CI 0.566–0.987, *p* = 0.040. No associations found for other micronutrients
Carr et al. [75]	Vitamin C	CRS	4101	12 wks GA; 24 wks GA	EPDS ≥ 13	12 wks GA and 24 wks GA	No assoc.

Mn*: micronutrient; Ax*: assessment.

**Table 8:** NOS assessments and micronutrients studied.

Author	NOS Score	Micronutrients Studied				
Rokoff et al., 2023 [72]	8					
Crayton et al., 2007 [73]	3					
Avalos et al., 2023 [55]	7		Folate			
van Lee et al., 2017 [58]	8		Folate			
Morris et al., 2020 [68]	5		Folate			
Blunden et al., 2012 [56]	9		Folate			
Lukose et al., 2014 [62]	8			Vitamin B12		
Chong et al., 2014 [64]	8		Folate	Vitamin B12		
Abou-Saleh et al., 1999 [60]	6		Folate	Vitamin B12		
Aishwarya et al., 2013 [59]	5		Folate	Vitamin B12		
Peppard et al., 2019 [63]	7		Folate	Vitamin B12		
Bodnar et al., 2012 [61]	8		Folate			
Basutkar et al., 2022 [49]	9				Iron	
Dama et al., 2018 [51]	8				Iron	
Hasdemir et al., 2022 [52]	8		Folate		Iron	
Ohsuga et al., 2022 [53]	7				Iron	
Albacar et al., 2011 [47]	8				Iron	
Armony-Sivan et al., 2012 [48]	8				Iron	
Chandrasekaran et al., 2018 [50]	8				Iron	
Lin et al., 2019 [45]	8				Iron	
Basutkar et al., 2021 [44]	9				Iron	
Evanchuk et al., 2024 [29]	9				Iron	
Jin et al., 2020 [74]	6					
Batalha et al., 2022 [65]	8			Vitamin B12		
Cruz-Rodriguez et al., 2024 [66]	8			Vitamin B12		
Dhiman et al., 2021 [67]	9			Vitamin B12		
Morris et al., 2019 [57]	8			Vitamin B12		
Carr et al., 2023 [75]	7					
Brandenbarg et al., 2012 [25]	7	Vitamin D				
Cunha Figueiredo et al., 2017 [27]	8	Vitamin D				
Huang et al., 2014 [30]	8	Vitamin D				
Woo et al., 2017 [39]	8	Vitamin D				
Accortt et al., 2021 [23]	8	Vitamin D				
King et al., 2022 [31]	7	Vitamin D				
Lamb et al., 2018 [32]	8	Vitamin D				
Nassr et al., 2022 [34]	7	Vitamin D				
Wang et al., 2023 [37]	7	Vitamin D				
Williams et al., 2016 [38]	8	Vitamin D				
Murphy et al., 2010 [33]	7	Vitamin D				
Uslu Yuvaci et al., 2020 [35]	7	Vitamin D				
Abedi et al., 2018 [21]	7	Vitamin D				
Accortt et al., 2016 [22]	7	Vitamin D				
Pillai et al., 2021 [46]	9	Vitamin D				
Jani et al., 2020 [40]	8	Vitamin D				
Fu et al., 2015 [77]	8	Vitamin D				
Noshiro et al., 2023 [41]	5	Vitamin D			Iron	
Bahramy et al., 2020 [42]	7	Vitamin D				
Al-Sabah et al., 2024 [43]	7	Vitamin D	Folate	Vitamin B12	Iron	
Roomruangwong et al., 2017 [10]	7					Zinc
Kurniati et al., 2020 [69]	4					Zinc
Indriasari et al., 2019 [79]	6					Zinc
Kavitha et al., 2021 [70]	8					Zinc
Wojcik et al. [71]	5					Zinc

**Table 9:** Quality assessment for RCTs as per JBI critical appraisal tool.

Author	JBI Quality Assessment
Paoletti et al., 2013 [54]	True randomisation was used to assign participants to two groups. Concealment and blinding of allocation were not made clear. The two groups were comparable in terms of age and BMI. Strict inclusion and exclusion criteria aimed to limit the effect of confounding factors. Treatment groups were treated identically, with the exception of intervention. Follow-up was complete.
Vaziri et al., 2016 [36]	Block randomisation was used to create roughly equal groups. The two groups were similar in terms of baseline demographic characteristics. It is not clear whether the investigators were blind or the participants were blind to the allocation. Losses to follow-up were described. Confounding factors were identified.
Dabbaghmanesh et al., 2019 [28]	Block randomisation was used. The study was double-blinded, with allocation concealed from all. The treatment groups were similar at baseline. Only a few confounding variables were described. Limited information was provided regarding follow-up and the difference between the groups.
Amini et al., 2022 [24]	True randomisation and double-blind allocation occurred. Treatment groups were comparable at baseline. Loss to follow-up was described. ANCOVA was used to adjust for confounding variables.
